# Repurposing existing drugs for COVID-19: an endocrinology perspective

**DOI:** 10.1186/s12902-020-00626-0

**Published:** 2020-09-29

**Authors:** Flavio A. Cadegiani

**Affiliations:** Adrenal and Hypertension Unit, Division of Endocrinology and Metabolism, Department of Medicine, Escola Paulista de Medicina, Universidade Federal de São Paulo (EPM/UNIFESP), Rua Pedro de Toledo 781 – 13th floor, São Paulo, SP 04039-032 Brazil

**Keywords:** COVID-19, SARS-CoV-2, ACE2, TMPRSS2, Pandemic

## Abstract

**Background:**

Coronavirus Disease 2019 (COVID-19) is a multi-systemic infection caused by the novel Severe Acute Respiratory Syndrome Coronavirus 2 (SARS-CoV-2), that has become a pandemic. Although its prevailing symptoms include anosmia, ageusia, dry couch, fever, shortness of brief, arthralgia, myalgia, and fatigue, regional and methodological assessments vary, leading to heterogeneous clinical descriptions of COVID-19. Aging, uncontrolled diabetes, hypertension, obesity, and exposure to androgens have been correlated with worse prognosis in COVID-19. Abnormalities in the renin-angiotensin-aldosterone system (RAAS), angiotensin-converting enzyme-2 (ACE2) and the androgen-driven transmembrane serine protease 2 (TMPRSS2) have been elicited as key modulators of SARS-CoV-2.

**Main text:**

While safe and effective therapies for COVID-19 lack, the current moment of pandemic urges for therapeutic options. Existing drugs should be preferred over novel ones for clinical testing due to four inherent characteristics: 1. Well-established long-term safety profile, known risks and contraindications; 2. More accurate predictions of clinical effects; 3. Familiarity of clinical management; and 4. Affordable costs for public health systems. In the context of the key modulators of SARS-CoV-2 infectivity, endocrine targets have become central as candidates for COVID-19.

The only endocrine or endocrine-related drug class with already existing emerging evidence for COVID-19 is the glucocorticoids, particularly for the use of dexamethasone for severely affected patients. Other drugs that are more likely to present clinical effects despite the lack of specific evidence for COVID-19 include anti-androgens (spironolactone, eplerenone, finasteride and dutasteride), statins, N-acetyl cysteine (NAC), ACE inhibitors (ACEi), angiotensin receptor blockers (ARB), and direct TMPRSS-2 inhibitors (nafamostat and camostat). Several other candidates show less consistent plausibility. In common, except for dexamethasone, all candidates have no evidence for COVID-19, and clinical trials are needed.

**Conclusion:**

While dexamethasone may reduce mortality in severely ill patients with COVID-19, in the absence of evidence of any specific drug for mild-to-moderate COVID-19, researchers should consider testing existing drugs due to their favorable safety, familiarity, and cost profile. However, except for dexamethasone in severe COVID-19, drug treatments for COVID-19 patients must be restricted to clinical research studies until efficacy has been extensively proven, with favorable outcomes in terms of reduction in hospitalization, mechanical ventilation, and death.

## Background

Coronavirus Disease 2019 (COVID-19) is a multi-systemic infection caused by the novel Severe Acute Respiratory Syndrome Coronavirus 2 (SARS-CoV-2), from the *coronaviridae* family. Some of the specific characteristics found in SARS-CoV-2, including long periods of incubation and shedding, large percentage of asymptomatic and pre-symptomatic infected subjects that may transmit the virus, and the prolonged resistance in surfaces [1–3], are plausible explanations for the inability to contain the SARS-CoV-2 spread, resulting in the current pandemic, although its mechanisms are still not fully elucidated, and may vary according to mutations that SARS-CoV-2 undergoes.

Prevailing symptoms in COVID-19 include anosmia, ageusia, dry couch, fever, shortness of brief, arthralgia, muscle soreness, fatigue, gastrointestinal symptoms, testicular and breast pain. However, slight mutations in the virus and variations in methodological assessments leads to heterogeneous clinical descriptions of COVID-19.

Besides age, data from regions with distinct epidemiological patterns consistently demonstrated that four other major factors are correlated with worse prognosis in SARS-CoV-2: uncontrolled diabetes, hypertension, obesity, and in-tissue exposure to androgens [3–9].

The unique SARS-CoV-2 characteristics and its unprecedented mechanisms of actions challenge the precise description of its mechanisms of actions and pathogenicity [10–15]. The only aspect of SARS-CoV-2 that has demonstrated undisputed characterization is its cell entry, which has shown to be dependent on angiotensin-converting enzyme-2 (ACE2) receptor and transmembrane serine protease 2 (TMPRSS2). While ACE2 is the site of coupling of the spike protein of SARS-CoV-2, TMPRSS2 primes the virus spikes and the ACE2 receptor, allowing its cell entry. The distribution of ACE2 expression in tissues is in fully accordance with the clinical manifestations of COVID-19, that has demonstrated to be multisystemic, although predominantly pulmonary, reinforcing the ACE2-centered hypothesis in COVID-19.

In addition, the vascular system, also largely affected in COVID-19, also induces pulmonary manifestations, since vascularity is critical for respiratory function. Conversely, to date, the only known regulators of TMPRSS2 expression are androgens, which may explain the prevailing presence of males in severe COVID-19, in particular those with androgenetic alopecia (AGA), in which TMPRSS2 expressed seems to be more expressed than in non-AGA males, due to increased hyperandrogenic hormones, specially 5-alpha dihydrotestosterone (5alpha-DHT), enhanced androgen receptor sensibility, or both [8, 9].

The understanding of the natural course of SARS-CoV-2 is imperative to provide hypotheses for potential therapeutic targets for COVID-19. Currently, COVID-19 can be divided into three stages, although further findings may lead to changes in the understanding of COVID-19 natural history of the disease. The first stage encompasses the period of SARS-CoV-2 viral infection, usually takes between five and ten days, and is dependent on ACE2 attached to surfaces, which seems to regulate the viral cell entry and infectivity, while free circulating ACE2 may preclude from viral infectivity by coupling with SARS-CoV-2 [16–19]. In this stage, since TMPRSS2 facilitates virus cell entry, its expression is also directly correlated with SARS-CoV-2 infectivity.

Unlike the first stage, common to all infected subjects, the second and third stages of COVID-19 are not obligatorily present, and depend on individual characteristics and predisposition. The second stage corresponds to the exacerbated inflammatory reactions to SARS-CoV-2. The second stage typically begins after the downregulation of membrane ACE2 due to its endocytosis that occurs in the first stage. Oppositely to what has been hypothesized for the first stage, increased attached ACE2 during the second stage is likely correlated with favorable outcomes, since ACE2 may limit the cytokine storm that underlies the Acute Respiratory Distress Syndrome (ARDS) in COVID-19.

In addition to the downregulation of attached ACE2 expression, the overexpression of the pro-inflammatory angiotensin II-angiotensin receptor type 1 (AT1) axis and the under-expression of the anti-inflammatory angiotensin 1–7 – G-coupled Mas receptor axis may contribute to the abnormal responses that lead to the cytokine storm, that determines the progression to the third phase, that corresponds to acute lung injury and related dysfunctions.

From the learnings on SARS-CoV-2 mechanisms of infection and disease, the complex relationship between ACE2 and SARS-CoV-2 has been demonstrated to be key to predict COVID-19 severity: while increased circulating ACE2 may provide protection by inhibiting SARS-CoV-2 coupling to attached ACE2, a dual correlation between lung membrane-attached ACE2 and COVID-19 has been demonstrated, since during the viral replication the ACE2 expression may enhance viral infectivity, whereas afterwards ACE2 becomes crucial to avoid over-inflammatory and over-immunologic responses, preventing the occurrence of ARDS.

### SARS-CoV-2: endocrinological and clinical risk factors related to endocrine-related disorders

While multiple mechanisms have demonstrated to participate in COVID-19 pathogenesis, the regulation of the Renin-Angiotensin-Aldosterone System (RAAS) has been demonstrated to be critical for the COVID-19 pathogenesis, since angiotensin 1–7 (Ang 1–7), ACE2 and AT1, that participate in the RAAS, are three of the four key modulators of SARS-CoV-2 infection patterns. The fourth key regulator of SARS-CoV-2 infectivity is TMPRSS2, which is largely and solely modulated by specific androgens, and depends on androgenic metabolomic profile and androgen receptor (AR) sensibility. In common, all major key mechanisms of SARS-CoV-2 interaction with organism are dependent on overall endocrine functions.

Abnormalities in the RAAS and ACE2 expression as being determinants of COVID-19 severity provide mechanistical substantiation for the overrepresentation of hypertension and obesity as risk factors for COVID-19. Conversely, TMPRSS2 overexpression may justify the higher occurrence of COVID-19 complications in males, particularly in those with androgenetic alopecia (AGA), in which TMPRSS2 is likely more activated compared to non-AGA males [8, 9]. In addition, up to 40% of patients with severe SARS-CoV-2 may present underlying silent congestive heart failure (CHF), leading to reduced pulmonary capacity and easier decompensation of cardiovascular and respiratory systems [20]. Together, dysfunctions in the RAAS, ACE2 and TMPRSS2 underlie all major risk factors for severe COVID-19.

In particular for obesity, the most remarkable risk factor in COVID-19, particularly among those below 50 y/o, multiple mechanisms have been proposed to justify the severity of COVID-19, including multiple sites of disruption in the RAAS system, ACE2 expression and activity, associated to an imbalance towards the hypertensive and pro-inflammatory angiotensin II- AT1 axis observed in obese subjects.

Since the RAAS, ACE2 and TMPRSS2 expression encompass virtually all tissues, and their biological actions are not restricted to endocrine regulation, one expects that manifestations related to their dysfunctional expression and activity would not be restricted to endocrine functions. Indeed, the pro-thrombotic state notably present during COVID-19, as well as hematological, kidney, hepatic, cardiovascular, neurological and gastrointestinal manifestations extensively reported in COVID-19 [21–47], are largely mediated by abnormalities in the RAAS, ACE2, and TMPRSS2.

In summary, the demonstration that overexpression of attached ACE2 compared to circulating, aberrancies in ACE2 expression and activity, predominant pro-inflammatory angiotensin II-AT1 over angiotensin 1–7-Mas receptor axis, and increased TMPRSS2 expression as keys to determine COVID-19 severity [48–53] allows the hypothesis that regulation of endocrine system may be central for improvement of COVID-19 related outcomes in clinical practice.

## Main text

### Strategies against SARS-CoV-2

The identification of effective treatments to reduce COVID-19 clinical outcomes, mortality and post-COVID manifestations is highly desired while definitive solutions like effective and safe vaccines are not universally available. Targets that address SARS-CoV-2 mechanisms of infection and risk factors allow proposals of more precise therapies to be potentially effective against COVID-19.

In the current lack of evidence on effective therapies, a major challenge is to identify or discover drugs that delivers high effectiveness, strong safety profile, and affordability for public health systems. In this regard, more than 400 novel drugs including more than 30 biological agents are undergoing clinical trials, among which some may show effectiveness, but will still lack long-term safety profile and is unlikely to be affordable for massive use.

Repurposing existing drugs for COVID-19 should be preferred over the development of new molecules due to four major reasons inherent to long used molecules [53–57]: 1. Well-established short- and long-term safety profile, risks, and contraindications, allowing directed monitoring and lower costs of follow-up and avoiding their use in formally contraindicated populations; whereas newly released drugs require longer studies, thorough monitorization and strict follow-up of special populations, due to the undetermined effects in large populations, since safety profile, detailed risk assessment and detection of uncommon adverse effects and complications can only be obtained in long-term large-scale studies; 2. Mechanisms of action tend to be better elucidated, allowing more precise predictions of clinical effects in COVID-19; 3. Clinicians are more likely familiarized with the clinical management of already existing drugs, including posology, effects, and complications, which is of great importance since the number of infected subjects does not allow COVID-19 to be managed within specialized centers; and 4. For COVID-19, patented drugs will unlikely have sufficient cost-effectiveness to justify their use in large scale, once the majority of infected subjects will cure without major clinical complications, irrespective of any treatment. Thus, the number necessary to treat (NNT) will be unconditionally high, which does not support expensive therapeutic options.

In this context, the clinical use prior to specific evidence of efficacy against COVID-19 has been accepted in the current lack of therapeutic options, particularly when risks of complications are high [55–57]. However, the *off-label* use, termed as compassionate, should be restricted to those steadily safe drugs, as learned from the harms caused by the unrestricted spread use of hydroxychloroquine [58] – which does not hamper from its potential effectiveness, particularly in the first stage of the disease, to be further elucidated.

Considering the current evidence, the employment of currently existing long used drugs should target SARS-CoV-2 infectivity, inflammatory response, or both, by addressing at least one of the following: ACE2 timing of expression in the lungs and balance between circulating and membrane-attached ACE2, enhancement of the angiotensin 1–7 axis, inhibition of TMPRSS2 actions, specific anti-inflammatory or immune-modulator effects, direct or indirect anti-viral activity, or blockage of harmful effects of dysfunctional RAAS overtly found in obesity.

In this context, endocrine targets have become central, as many of the drugs for the prevailing endocrine or endocrine-related dysfunctions, including anti-diabetics, anti-hypertensives, hormones and have demonstrated effects on one or more sites of actions in COVID-19, once SARS-CoV-2 infectivity and response are highly modulated by different endocrine pathways with strong interactions.

The characteristics to be present within proposed drugs should include well-established risks and contraindications, cardiovascular safety or protection, hematological and vascular safety or protection, with neutral or preventive effects of thromboembolic events, favorable effects on the RAAS and ACE2, neutral or downregulation of the androgen-mediated TMPRSS2, and inhibition or non-exacerbation of acute lung injuries. Drugs that address subclinical CHF and other cardiac dysfunctions may provide independent additional protection, since CHF and elevated cardiac markers are highly prevalent among hospitalized COVID-19 patients [59–61].

A systematic search of the following terms have been searched in PubMed database: “(name of the drug)” or “(name of the drug class)” or “(hormone)” + “COVID” or “SARS-CoV-2” or “lung injury” or “ARDS” or “viral” or “renin-angiotensin system” or “renin-angiotensin-aldosterone system” or “TMPRSS2” or “ACE2” or” RAAS”. In clinicaltrials.gov, search was performed using the expressions “COVID” + “(name of the drug)” or “(name of the drug class)” or “(hormone)”.

Candidates for COVID-19 have been ordered according to their likelihood to provide protection for COVID-19: 1. Of major relevance: more likely to provide clinical benefits, with preliminary or consistent clinical data on COVID-19; 2. Of moderate relevance: strong plausibility but weak evidence on COVID-19; 3. Of minor relevance: moderate plausibility but solely theoretical; 4. Discourage use in COVID-19: those that harmful effects may overtake benefits on COVID-19; and 5. A summary of drugs unrelated to the endocrine system with potential benefits for COVID-19.

Data on each drug class as candidate for COVID-19 has been presented following a specific logical sequence: 1. Mechanisms of action that could theoretically provide benefits for COVID-19; 2. How the drug could be used to treat COVID-19, including the target stages of the disease; and 3. Current specific data on COVID-19, if any.

### Candidate drugs against COVID-19

Several different drugs have elicited hypothetical benefits against COVID-19, including hormones, anti-diabetics and anti-androgens, although the vast majority remains only theoretical, and many of these drugs could provide protection for their regular users, but not necessarily show clinical benefits in COVID-19 if specifically used for this purpose.

#### Of major relevance

The only endocrine-related drug class that currently has evidence for COVID-19 are the glucocorticoids. Their use for severe illnesses has dubious and contradictory data [62, 63], that seems to depend on the etiology and patterns of lung injury, level of severity, and level of contribution of an overreactive inflammatory response for the severe state, since the two major actions expected from glucocorticoids are their strong anti-inflammatory properties and as enhancers of the physiological response to stress. However, specifically for severe COVID-19, emerged data has been favorable for the use of glucocorticoids, since it has been demonstrated to reduce mortality among hospitalized patients, particularly those in mechanic ventilation [64], which corresponds to the third stage of COVID-19, and 10 currently ongoing clinical trials are testing glucocorticoids in this stage [65]. Conversely, for mild to moderate COVID-19, although the use of corticoids was initially discouraged due to potential delay of viral clearance and increase of viral infectivity [66–68], improvements have been reported when used during the second stage of COVID-19, before the development of ARDS [69–74], likely due to glucocorticoid ability to prevent cytokine storm. Since the majority of glucocorticoids has concurrent mineralocorticoid effects, *i.e*, aldosterone-like actions [75–79], glucocorticoid may enhance the RAAS and mimic detrimental effects of hypertension and obesity in this system, potentially increasing the risks of complications related to COVID-19.

Considering the differences in the characteristics of each corticoid, the specificity of glucocorticoid over mineralocorticoid action when selecting the corticoid to be used should not be despised. In particular, dexamethasone exerts powerful and highly selective glucocorticoid effects, and together with betamethasone, they are the only exogenous corticoids with no mineralocorticoid actions [75–79]. Strong glucocorticoid with absence of mineralocorticoid actions which should be the preferred corticoid regimen for COVID-19, since mineralocorticoid activity may indirectly stimulate viral spread through imbalance between circulating and membrane-attached ACE-2, and deteriorate cardiac and pulmonary functions, central in COVID-19. This may explain the superior efficacy of dexamethasone in severely ill patients with COVID-19 [64], although direct comparisons with other glucocorticoids have not been performed.

In summary, glucocorticoids have potential benefits when used in the second stage and demonstrated benefits in the third stage of COVID-19.

#### Of moderate relevance

Some drug classes have moderate relevance as candidates for COVID-19 due to their strong plausibility yet weak or null evidence for COVID-19.

Males have been shown to be overrepresented among those severely affected by COVID-19, which remained significant after adjustments for age, body mass index (BMI), and presence of comorbidities [80, 81]. This consistent observation finds plausibility in the active participation of the androgen-driven TMPRSS2 to facilitate SARS-CoV-2 cell entry. However, this correlation is more complex, as young males had better outcomes compared to BMI- and disease-matched older ones, despite having higher testosterone levels, Possibly, intracellular conversion from T into more androgenic hormones, particularly 5alpha-dihydrotestosterone (5alpha-DHT), may better drive SARS-CoV-2 infectivity, which is supported by the observation that bald men, who typically have higher intracellular DHT levels, are at higher risk of developing severe COVID-19 than their non-bald counterparts [82, 83].

Anti-androgenic approaches intuitively seem to be protective from COVID-19 in males. Indeed, prostate cancer patients receiving androgen-deprivation therapies (ADT) appear to be partially protected from SARS-CoV-2 infections [84, 85]. Besides mitigating TMPRSS2 expression [86], androgen-deprivation or antagonizing therapies may suppress the RAAS overexpression induced by androgens, which can be observed in post-pubertal males and hyperandrogenic states in females [87–89].

Anti-androgenic therapies encompass those that inhibit the hypothalamic-pituitary-gonadal axis, including modulators of the gonadotrophic inhibitory hormone (GnIH) and Kisspeptin-Kiss1receptor axis and gonadotrophic releasing hormone (GnRH) agonists (leuprolide, goserelin, triptorelin) and antagonists (degarelix), androgen receptor (AR) inhibirors (ARi - cyproterone, spironolactone, eplerenone, flutamide) and 5alpha-reductase inhibitors (finasteride, dutasteride). Among these, AR and 5alpha-reductase inhibitors deserve attention, since they provide prompter anti-androgenic actions and some present additional anti-COVID-19 properties.

The major representatives of ARi are spironolactone and eplerenone, that also act mineralocorticoid receptor (MR) antagonists, inhibiting aldosterone actions, which represents the bioactive RAAS end-product [90–92]. From these, spironolactone is the most widely commercially available, has an extensive safety profile, is an effective anti-hypertensive, and has demonstrated ability to protect and prevent damage in the heart and kidneys [93–97].

In addition to the protection reported to be provided by spironolactone, specific actions against SARS-CoV-2 actions have been proposed, including increased availability of free circulating ACE2 in response to a hyperreninemic state induced by MR antagonism [98–104], reduction of TMPRSS2 expression due to antagonism of AR [105–107], reversal of RAAS abnormalities induced by obesity [108, 109], and possible direct anti-inflammatory and anti-viral actions that hamper lung injuries [110–121]. There are currently three ongoing clinical trials with spironolactone [122–124].

Dutasteride and finasteride are the two major 5alpha-reductase inhibitors used in clinical practice and studied for safety and effective profile in the long run [125–127]. The rationale for their use is based on the blockage of conversion of testosterone into 5alplha-DHT and mitigation of TMPRSS2 expression [128–130], eventually hampering the overrepresentation of males, particularly bald ones, in severe COVID-19. Their benefits may be exhibited if used as a preventive strategy or during the first stage of COVID-19, and has demonstrated correlations with lower severity, although causality could not be established [131, 132]. There is one clinical trial currently testing dutasteride in COVID-19 [133].

Statins (simvastatin, atorvastatin, rosuvastatin, pitavastatin) are inhibitors of the 3-hydroxy-3-methylglutarul-coenzyme A (HMG-CoA) reductase that act primarily as antilipemic agents, with extensive efficacy against cardiovascular events, and pleiotropic anti-inflammatory, antithrombotic, anti-oxidative, immunomodulatory, antiarrhythmic, and direct anti-atherogenic effects [134–136]. From these pleiotropic actions, statins have been purposed to reduce the occurrence and severity of ARDS states and the effects of endotoxin in lung injury [137–141], acting against COVID-19, particularly in the second and third stages [104, 142–145], in addition to the effects on the RAAS, including the decrease of angiotensin II synthesis and action, and reduction of the RAAS-induced oxidative state [146–148]. There are currently five clinical trials [149–153] and one observational study [154] with statins for COVID-19.

Vitamin D is an actual hormone with calcium metabolism, immunologic and metabolic actions. Preliminary observations have correlated vitamin D levels and outcomes in COVID-19, allowing hypotheses on vitamin D as being protective from COVID-19 due to its potential benefits antiviral activity [155–167], attenuation of lung injuries [168–173], and possible slight suppressive although inconsistent effects on RAAS [174–177], and neutral effects on TMPRSS2 [178, 179], being a potential candidate to protect from SARS-CoV-2 infectivity, i.e., during its early stage, although only observational studies have been published to date. Despite preliminary reports on hypothetical vitamin D actions on decreasing lung injury severity, this is still only theoretical, and should not be considered as a strong candidate for second and third stages of COVID-19. Currently, 31 studies are evaluating vitamin D supplementation and status in COVID-19, alone or in combination with other therapies [65].

Although used as a nutritional supplement, N-acetyl cysteine (NAC), a precursor of L-cysteine, enhances glutathione elevation biosynthesis and acts as a direct scavenger of free radicals, particularly reactive oxygen species (ROS) [180, 181]. NAS has demonstrated robust antioxidant effects both in vitro and in vivo, and has been successfully employed in a variety of diseases, with emerging evidence in polycystic ovary syndrome (PCOS), fertility abnormalities, chronic inflammation, particularly colitis, acetaminophen intoxication, asthma and neurodegenerative disorders [182]. NAC exerts multiple effects on the modulation of the inflammatory and immunologic responses, including inhibition of the inflammasome pathways [interleukin-1β (IL1β), IL18, and tumor necrosis factor-ɑ (TNFɑ)], increase of T cells activity, and improvement of redox status, particularly under intense oxidative stress [183–187], has shown ability to diminish acute lung injuries [188], and may impair ACE2 actions when coupled with SARS-CoV-2 [189, 190]. Collectively, these mechanisms convey the hypothesis of NAC as a strong candidate against COVID-19 [191, 192], in particular for the second stage, aiming to prevent progression to ARDS, and is undergoing six specific clinical trials [193–198], among which five aim to prevent the occurrence of third stage in COVID-19.

Aspirin is a potent suppressor of prostaglandins and thromboxane A2 (TXA2) generation due to its irreversible inactivation of the cyclooxygenase (COX) enzyme, yielding anti-inflammatory and anti-thrombotic effects, respectively [199]. In addition of its regular use to prevent cardiovascular disease in those at high risk, aspirin may prevent gastrointestinal tract cancers [200–202] and participate in a wide range of different disorders [203], although its effects are highly dependent on the timing and dose administered [204]. While low doses aspirin may play indirect beneficial effects in the RAAS, including suppression of angiotensin II actions, high doses (> 200 mg/day) may hamper cardioprotective and lung-protective effects of the majority of drugs that address RAAS [205–207]. In the lungs, AAS may confer protective effects on the severity of lung injury induced by any endotoxin, and also lower the risk of ARDS, in particular in those previously using aspirin [207–209]. Because of these mechanisms, aspirin has been proposed to protect from COVID-19 during its second and third stages, in special under severe manifestations, and is being tested in ten clinical trials in COVID-19, all aiming to prevent or treat severely ill patients [65]. Other antithrombotic agents, in special the direct inhibitors of factor Xa, apixaban and rivaroxaban, have also demonstrated ability to attenuate lung injury [210, 211], could be potential candidates for the second and third stages of COVID-19, and rivaroxaban is being currently tested for COVID-19 in four clinical trials [212–215].

Although the anti-hypertensive classes of ACE inhibitors (ACEi) and angiotensin receptor blockers (ARB) have been initially correlated with worse outcomes in COVID-19 due to potential SARS-CoV-2 infectivity by the increase of lung membranse-attached ACE2 expression [11, 216, 217] and preliminary observations that hypertensive patients treated with ACEi or ARB could be at higher risk to develop ARDS and require mechanical ventilation [3, 218–220], not only these correlations have found no corresponding data on larger trials [221, 222], but they have been proposed to be protective, once their direct actions in the RAAS may be clinically helpful during the second stage, in which increased lung membrane ACE2 expression is crucial to prevent cytokine storm, for the balance between angiotensin II and 1–7, and to reduce COVID-19 induced ARDS [223, 224] either if introduced in the second stage of COVID-19, or among those chronic users. The controversy on ACEi, ARB and COVID-19 still remains, including five clinical trials still evaluating whether the use of ACEi and ARB is harmful [124, 225–228], whereas more than 20 clinical trials are testing these classes to reduce COVID-19 severity [65].

In addition to the anti-androgen actions of androgen inhibitors and AR antagonists aiming to reduce TMPRSS2 expression, direct TMPRSS2 blockers through serine protease inhibition have been proposed as potential drugs against COVID-19, including nafamostat, camostat, bromhexine, plasma alpha-1-antitrypsin, leupeptin [229–231]. Among these, nafamostat, a short-action anti-thrombotic with antiviral activity, and camostat have been proposed as treatment options for COVID-19, despite their high costs [230, 231]. There are currently three and eight clinical trials testing nafamostat [232–234] and camostat [235–242], respectively.

#### Of minor relevance

Drug classes and hormones with merely theoretical plausibility have been listed as of minor relevance for COVID-19.

While males, in particular those affected by androgenetic alopecia (AGA), have been correlated with worse prognosis in COVID-19, this could be explained by AR sensibility and DHT concentrations, rather than testosterone per se, since young males, with the highest testosterone levels, are not at higher risk when compared to age-matched young females. Indeed, on the opposite direction, one single study suggested that with lower testosterone concentrations could predict worse outcomes in COVID-19 [243], although low testosterone is more likely a consequence than a cause of COVID-19 severity, since its acute reduction due to suppression of the hypothalamic-pituitary-gonadal (HPG) axis may be directly correlated with level of severity, the hypothalamic hypogonadism that typically occurs in metabolic and inflammatory diseases, including cardiovascular and obesity, corresponds with those at higher risk for severe COVID-19 manifestations, which means that hypogonadotrophic hypogonadism and severe COVID-19 are both consequences of a same root cause (inflammatory and metabolic diseases), and indicates that testosterone could be potentially used as an indirect marker, but not necessarily as a candidate as a therapeutic agent for COVID-19.

A potential use of testosterone as a muscle anabolic agent for those recovering from severe COVID-19 could be a matter of discussion, since COVID-19 leads to a sort of muscle hypercatabolic state that leads to disproportionate muscle loss and consequent difficulties in performing basic personal activities, such as raising arms to eat, walking, or even inspiring, in a worse extent than the expected for the time spent in unconscious and in mechanic ventilation. However, while proposed and tested for other cachectic states [244–246], there are no reports on testosterone use for muscular recovery after COVID-19 or ongoing clinical trials on COVID-19.

Estrogens have demonstrated beneficial actions against viral infections and respiratory complications, as clinically observed by better outcomes in women during reproductive age [159, 247–259], due to their protective effect on endothelial function, vasodilation in the pulmonary vasculature, stimulation stimulate of the humoral response to viral infections [159, 247–253], and modulation of inflammatory responses [248, 249], leading to improved outcomes in acute lung injuries of any etiology [254–259]. Estrogens favorably modulates the RAAS in females [260–262], whereas the androgen-mediated TMPRSS2 expression has dual correlation with estrogens [248, 263, 264].

However, while COVID-19 has been extensively correlated with thrombotic events of different natures through a range of underlying mechanisms [265–267], and has become a major player in the COVID-19 pathogenesis [267], estrogens have been historically correlated with increased thromboembolic events, which could be a limiting argument for its use in COVID-19. Nonetheless, while endogenous estradiol is only correlated with this nature of events when associated with increased free testosterone and decreased se hormone binding globulin (SHBG) [268], the correlation of exogenous estrogens and thromboembolism is largely justified by the route of administration, orally administered estrogens lead to increased hepatic production of pro-coagulants induced by its first liver first-passage effect, that does not occur in non-oral regimens. Indeed, large observational studies and a meta-analysis have shown no increased risk of thromboembolism among women taking non-oral estrogen replacement therapies [269–271].

Collectively, the prevailing possible protective effect of estradiol against COVID-19 indicates this as a potential therapeutic target for the first and second stages of COVID-19 to prevent more severe complications, although oral regimens must be avoided to prevent synergistic effects with the pro-thrombotic stage inherent to COVID-19.

Type 2 diabetes mellitus (T2DM) has been recognized as a major independent risk factor for severe COVID-19, while the level of glucose control on T2DM may be one of the drivers of severity of COVID-19, particularly before hospitalization, through glucose and non-glucose mediated [272–274]. Overall, in addition to the glucose lowering effects, anti-diabetic drugs may exhibit additional pleiotropic effects specific to each class, that offer unspecific and viral-specific protection patterns, and have been hypothesized as potential agents against COVID-19.

Metformin, the first-line therapy for T2DM with undisputed efficacy and safety and additional antineoplastic, antiaging, anti-inflammatory, immunomodulatory, cardio-, neuro-, hepato-, and nephroprotective actions [275, 276], has been proposed as a multi-action protection drug candidate for COVID-19 [277–283], particularly due to its strong systemic reparatory and modulatory mechanisms. While its effects on RAAS seem to be neutral, lung injury can be efficiently prevented and relieved by metformin, specially by the promotion of microvascular repairing actions [284–289], and has also exhibited antiviral activity, enhanced lymphocyte B function and enhanced innate immunity [290–294]. Conversely, metformin has formal contraindication for severe conditions due to the risk of lactic acidosis [295]. For these reasons, metformin could be a candidate for the second stage of COVID-19, before the development of severe respiratory manifestations, although no specific clinical trials are being currently conducted.

Sodium-glucose Co-transporter 2 inhibitors (SGLT2i) (dapagliflozin, empagliflozin, canagliflozin, ipragliflozin, ertugliflozin) are a newly developed anti-diabetic drug class that promotes glycosuria through inhibition of renal glucose reabsorption, alleviating hyperglycemic states. Unexpected improvements of cardiovascular events, overall mortality, liver metabolic dysfunctions, kidney function, and pancreas activity observed in larger and longer randomized clinical trials (RCTs) and real-life studies were not completely justifiable by its glucose-, body weight-, and blood pressure-lowering effects [296–298]. Although effects on overall viral replication or lung injury are yet to be unraveled, SGLT2i’s have shown active suppression of the overall RAAS due to the negative water balance induced by its concurrent natriuresis, and additional selective mitigation of the angiotensin II-AT1R axis [299–302]. Among SGLT2i, dapagliflozin has been proposed as a protective tool against COVID-19, particularly in the first and second stages, and is currently undergoing two specific clinical trials [303–305].

Analogues of the glucagon-like peptide-1 (GLP-1) class B G-protein-coupled receptor (GLP-1Ra) (lixisenatide, liraglutide, exenatide, dulaglutide, semaglutide) have been first developed to act as glucose-lowering agents. GLP-1Ra have further demonstrated to address obesity, neurodegenerative disorders, have elicited a range of pleiotropic actions due to the wide and heterogeneous distribution of GLP-1R, and have shown to reduce major cardiovascular events, which, cannot be fully justified by improvements in glucose and body weight. GLP-1Ra may exert beneficial effects by controlling glucose levels during infection [306], possibly attenuating lung injury under different circumstances - at least in animal models [307–309] - and restoring lung function in ARDS [310]. GLP-1Ra have also favorable effects on the RAAS by inhibiting angiotensin II while maintaining circulating ACE2 levels [311–315]. Hence, GLP-1Ra has theoretical potential to act in the second stage against COVID-19, although there is no currently ongoing clinical trials for COVID-19.

Dipeptidyl peptidase-4 inhibitors (DPP4i) are anti-diabetic drugs that act indirectly enhances incretin hormone actions in a diffuse manner, leading to positive actions in the inflammatory, immunologic, and vascular systems, and consequently has been proposed to be potential candidates against COVID-19 [306, 316–318], currently being tested in two clinical trials. Besides the attenuation of angiotensin II activity [318], DPP4i may also prevent acute lung injury in response to different stressors [319–324] and has shown inhibitory effects on other coronaviruses, including the Middle East Respiratory Syndrome Coronavirus (MERS-CoV) [325–327], which could play protective role in the first and second stages of COVID-19 [328]. Currently, there are four clinical trials testing DPP4i for COVID-19, including two with sitagliptin and two with linagliptin [329–331].

Thiazolidinediones, also termed as glitazones, currently represented by pioglitazone, are nuclear receptor peroxisome proliferator-activated receptor gamma (PPARγ) and partial PPARα agonists with anti-diabetic and other beneficial metabolic properties. Once PPAR-α and -γ agonism exerts multiple metabolic, inflammatory and immunologic benefits, pioglitazone has been proposed as a candidate against COVID-19 [282, 332], despite the lack of current clinical trials to date. Pioglitazone may exert beneficial effects in the RAAS, including marked raise of serum ACE2 levels, which couples with SARS-CoV-2 and preclude viral coupling with attached ACE2 and consequent cell entry, and substantial increase of angiotensin- [1–6] and angiotensin-2 receptor (AT2) concentrations [333–336], undermining angiotensin II actions, may abolish acute lung injury by acting in a range of actions, including positively modulation of macrophage activity, reduction of neutrophil recruitment in response to endotoxin, and reduction of inflammation during sepsis [337–341], and also has direct anti-viral effects [342–345]. Due to its multiple pleiotropic effects, glitazones could theoretically be candidates for all stages in COVID-19 [332], and is currently being tested in one clinical trial [346].

Isotretinoin, a 13-cis-retinoic acid, is a drug extensively used to treat moderate-to-severe acne with vitamin-A like actions, while its metabolites act as retinoic acid receptor (RAR) and retinoid X receptor (RXR) agonists, with pro-apoptotic effects, although its exact mechanisms of actions are not fully elucidated [347, 348]. Despite the lack of reports on prevention or attenuation of lung injury, viral replication, specific immunologic or anti-inflammatory actions, and meaningful actions in the RAAS, isotretinoin has been proposed for the second stage of COVID-19, de due to its anti-inflammatory and immunomodulatory effects, and is being tested in five clinical trials for COVID-19 [349–353].

Rimonabant acts in the endocannabinoid (CB) system, a highly preserved mammalian system that exerts ubiquitous and diverse regulatory actions, including those in metabolism, central nervous system (CNS), inflammatory and immunologic pathways, as a selective CB-1 (cannabinoid receptor subtype 1) antagonist. Rimonabant has been first approved for obesity due to its strong anorexigenic effects [354, 355], but an unacceptable suicide rate has been detected during its post-market clinical trial (CRESCENDO), being withdraw from the market [356]. However, learnings from the CB system encouraged further investigations for the development of improved drugs without psychiatric effects, and has demonstrated mitigation of low-grade inflammation typically observed in obesity [357, 358], beneficial effects on the RAAS [359, 360], and immunomodulation [361–363], which allowed to propose rimonabant as a candidate for first and second stages in COVID-19 [364, 365], although it could only be tested for strict experimental purposes, since it has been banished since 2010.

Phosphodiesterase 5 (PDE5) inhibitors (PDE5i), including sildenafil, tadalafil, vardenafil and avanafil, are drugs that block the cyclic GMP-specific phosphodiesterase type 5 located in smooth muscle cells, that consequently line vessels in a variety of tissues, leading to tissue-specific vasodilatations. Sildenafil, the first PDE5i, has been first approved to treat erectile dysfunction [366], and has been further extended to primary pulmonary hypertension. In the RAAS, sildenafil has shown to enhance circulating ACE2 concentration and to exert dual effects on the angiotensin II-AT1-angiotensin 1–7-AT2 balance, with uncertain effects on COVID-19 [367], while sildenafil may provide protective effects for acute lung injuries due to its abilities to inhibit neutrophilic actions within the lungs and release of pro-inflammatory cytokines, reduce oxidative stress, inhibit apoptosis of epithelial cells, prevent lung edema formation, and improve respiratory parameters [368–370]. Positive effects of sildenafil treatment have also been observed with its effects on immunomodulation, angiogenesis, and platelet regulation [370]. The potential benefits of sildenafil on respiratory, inflammatory, vascular, and immunologic parameters based its proposal as a potential drug against COVID-19 [371] that would hypothetically protect in second and third stages, and is being tested in one clinical trial [372].

#### Discourage use in COVID-19

Some drug classes not only may show few or no benefits for COVID-19, but may also exhibit deleterious effects that may overcome specific benefits, if any.

While estrogens may present protective effects from COVID-19, their actions in alpha and beta ERs are more complex. Specific selective estrogen receptor modulators (SERMs), including tamoxifen and raloxifene, could theoretically be an additional target to be attempted in COVID-19, despite the lack of any specific data. While raloxifene has been researched for systemic actions, showing to be neutral in the RAAS [373, 374] potentially protective against lung injury [375], and neutral in the cardiovascular system [376], tamoxifen may increase risk of thromboembolic events and stroke when administered orally [377], and does not present any non-oral formulation. Despite the increased risk for thrombosis, which is particularly concerning in the pro-thrombotic state of COVID-19, tamoxifen, not raloxifene, is being currently studied in one clinical trial [350].

Aromatase inhibitors, including anastrozole, letrozole, and exemestane, mitigate testosterone conversion into estrogens, as per its inherent mechanisms of action. Aromatase inhibitors have exhibited harmful effects in the RAAS [378] and indirectly increase of 5alpha-reductase activity and DHT levels [379, 380], enhancing TMPRSS2 expression. Since these effects in the RAAS and TMPRSS2 may potentialize SARS-CoV-2 infectivity and increase risk of thromboembolic events, aromatase inhibitors should be discouraged as candidates for COVID-19.

Unlike estrogens, progestogens lack demonstration of any mechanism of protection from COVID-19, has suppressive effects on both innate and cell-mediated immune responses, in particular inducing T-lymphocyte cell death [381], apparently do not have any major effect in the RAAS [382–384], although hormones with progesterone activity compete with aldosterone in the MR. Because of the suppressing effects on T-lymphocyte, progesterone should be discouraged for COVID-19, despite being currently evaluated for COVID-19 in hospitalized men [385].

#### Drugs unrelated to the endocrine system

The present review focused on the potential of endocrine drugs and targets as candidates to protect from COVID-19. However, non-endocrine drugs have shown strong effect and evidence as direct or indirect anti-viral activity [386]. Drug classes unrelated to the endocrine system that have been proposed and are being currently tested for COVID-19 include antiviral drugs (lopinavir/ritonavir, remdesivir, darunavit/umifenovir, favipiravir, nelfinavir and oseltamivir), broad-spectrum anti-parasitic drugs (nitazoxanide, ivermectin), antimalarics (mefloquine, chloroquine and hydroxychloroquine), the anti-alcohol addiction drug disulfiram, and anti-inflammatory drugs to modulate immunologic response, including interferons, tocilizumab, and other biological molecules and monoclonal antibodies (baricitinib, sunitinib – AAK1/GAK inhibitors, upadacitinib, tofacitinib – JAK inhibitors, and belinostat – HDAC inhibitor) [386].

### Final discussion

**Fig. 1:**
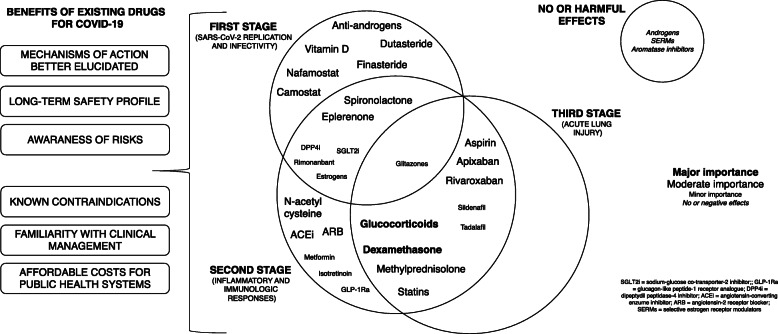
Potential endocrine targets, their respective candidate drugs, and benefits of repurposing existing drugs for COVID-19

Figure 1 summarizes the theoretical potential candidates according to the stages of COVID-19 that they may provide benefits, as well as the list of reasons the support the testing of existing drugs for COVID-19.

Collectively, current understanding shows that SARS-CoV-2 infection is enhanced by abnormally high and low attached and circulating ACE2 expression respectively, increased pro-inflammatory angiotensin-II-AT1 axis, reduced anti-inflammatory angiotensin- [1–6]-Mas receptor axes, and increased TMPRSS2 activity. These abnormalities are able to justify obesity, hypertension, and AGA males as being major risk factors of COVID-19 complications. Multiple endocrine-related drugs, including hormones, anti-diabetics, anti-androgens and other types of molecules exhibit actions in one or more sites that may inhibit SARS-CoV-2 infectivity, replication, or inflammatory or immunologic overreaction and consequent ARDS.

Except for dexamethasone in severe COVID-19, while effective and safe drugs for SARS-CoV-2 lack, researches should be encouraged to consider testing existing drugs with well-established safety profile, known risks and contraindications, notorious clinical management, favorable cost-effectiveness, and promising results in COVID-19. Specific combination of some of the candidates for COVID-19 may exhibit synergistic effects for COVID-19, and should also be considered for clinical trials.

Besides tested in clinical trials for COVID-19, drug classes listed as candidates for COVID-19 should also be evaluated in patients using these drugs regular- and chronically for original purposes, before COVID-19 is installed, since acute and chronic use of overall drugs may present distinct effects [387].

Finally, it must be emphasized that regardless of theoretical potential to protect from COVID-19 or preliminary favorable outcomes, drug treatment for COVID-19 patients must be prescribed only after consistent demonstration of efficacy in randomized clinical trials. After proven efficacy, use of drug must be restricted for patients in the specific stage of COVID-19 for which drug has demonstrated efficacy, since drugs can lead to opposite results, as demonstrated with dexamethasone, which while reduced mortality in critically ill patients, subgroup analysis suggested that its use in mild and non-hospitalized patients led to increased mortality [64]. Drugs must prove efficacy in terms of reduction of hospitalization, need of intensive care, mechanical ventilation, and death**,** and prevention of long-term pulmonary, musculoskeletal, and other physical and mental consequences, in order to be clinically used.

## Conclusions

In the current lack of solid evidence for any specific drug against COVID-19, researchers should consider testing existing drugs with robust long-term safety profile, absence of major risks or life-threatening complications, known contraindications, familiarity in medical community and health care, and plausibility to exhibit protective effects from COVID-19. However, it is mandatory that these drugs are only prescribed in case their efficacy has been proven in clinical trials, and specifically for those patients in the COVID-19 stage for which efficacy has been proven.

## Data Availability

There is no additional data than the contained within the present manuscript.

## Abbreviations

ACE2: Angiotensin-converting enzyme-2
AT1: Angiotensin receptor-1
AT: Angiotensin receptor-2
CHF: congestive heart failure
COVID-19: Coronavirus Disease 2019
RAAS: Renin-Angiotensin-Aldosterone System
SARS-CoV-2: Severe Acute Respiratory Syndrome Coronavirus 2
TMPRSS2: Transmembrane serine protease 2
